# Arthrofibrosis after total knee arthroplasty: patients with keloids at risk

**DOI:** 10.1186/s10195-020-00563-7

**Published:** 2021-01-05

**Authors:** Travis R. Flick, Cindy X. Wang, Akshar H. Patel, Thomas W. Hodo, William F. Sherman, Fernando L. Sanchez

**Affiliations:** Department of Orthopaedic Surgery, Tulane University School of Medicine, 1430 Tulane Avenue, New Orleans, LA 70112 USA

**Keywords:** Total knee arthroplasty, Keloid, Arthrofibrosis, Manipulation under anesthesia, Lysis of adhesions, Revision, Reoperation, Complications, Clinical outcomes

## Abstract

**Background:**

Arthrofibrosis remains one of the leading causes for revision in primary total knee arthroplasty (TKA). Similar in nature to arthrofibrosis, hypertrophic scars and keloid formation are a result of excessive collagen formation. There is paucity in the literature on whether there is an association between keloid formation and the development of arthrofibrosis following TKA. Therefore, the purpose of this study was to utilize a large nationwide database to identify and compare the rates of postoperative complications related to arthrofibrosis after primary TKA in patients with history of hypertrophic scar and keloid disorders versus those without.

**Methods:**

Patient records from 2010 to the second quarter of 2016 were queried from an administrative claims database, comparing rates of arthrofibrosis, manipulation under anesthesia (MUA), lysis of adhesions (LOA), and revision TKA in patients with chart diagnosis of keloids versus those without in patients who underwent primary TKA. Data analysis was performed using R statistical software (R Project for Statistical Computing, Vienna, Austria) utilizing multivariate logistic regression, chi square analysis, or Welch’s *t*- test where appropriate with *p* values < 0.05 being considered statistically significant.

**Results:**

Of 545,875 primary TKAs, 11,461 (2.1%) had a keloid diagnosis at any time point in their record, while 534,414 (97.9%) had not. Patients in the keloid cohort had a significantly higher association with ankylosis within 30 days (OR, 1.7), 90 days (OR, 1.2), 6 months (OR, 1.2), and 1 year (OR, 1.3) following primary TKA. The keloid cohort also had a significantly greater risk of MUA (90-day OR, 1.1; 6-month OR, 1.1; 1-year OR, 1.2) and LOA (90-day OR, 2.2; 6-month OR, 2.0; 1-year OR, 1.9).

**Conclusion:**

Patients with keloids have increased odds risk of arthrofibrosis following primary TKA. These patients are subsequently at a higher odds risk of undergoing the procedures necessary to treat arthrofibrosis, such as MUA and LOA. Future studies investigating confounding factors such as race, prior surgery, range of motion, and postoperative recovery are needed to confirm the association of keloid diagnosis and arthrofibrosis following primary TKA demonstrated in this study.

**Level of Evidence:**

Level III retrospective comparative study.

## Introduction

Total knee arthroplasty (TKA) has emerged as one of the most frequently performed and successful surgeries in orthopedics today with over 95% survival at 15 years [1, 2]. Studies project the incidence of TKA will grow by 85% between 2014 and 2030 from 935,000 to 1.26 million procedures annually [3, 4]. Patient satisfaction with the surgery has been reported as good to excellent with decreases in pain and increased functionality in 70–90% of patients [5–8]. While TKA has proven to be successful, one of the leading causes of failure and hospital readmission is arthrofibrosis [9–11], with recent studies indicating arthrofibrosis being the primary surgical indication for revision in 4.5% of TKAs being performed annually [12].

The etiology of arthrofibrosis is multifactorial with known risk factors being previously identified which result in an excessive amount of fibrous scar tissue production in the joint, such as smoking, systemic disease, history of previous surgeries, limited preoperative range of motion, complexity of the TKA surgery, and poor postoperative rehabilitation [9, 13]. This complication can be debilitating to the patient as the excessive scar tissue restricts range of motion and causes pain, reducing the patient's quality of life [14, 15]. In addition, patient-reported stiffness from arthrofibrosis often results in surgical intervention such as manipulation under anesthesia (MUA), consisting of a surgeon manipulating the knee through full range of motion while the patient is sedated, lysis of adhesion (LOA), which consists of using an open or arthroscopic approach to access and debride adhesions, and revision TKA surgery [10, 11, 13].

Similar in nature to arthrofibrosis, hypertrophic scars and keloid formation are a result of excessive collagen formation [16, 17]. Additionally, these disorders most commonly occur after an inciting stimulus such as an incision into the dermis for keloids and a TKA for arthrofibrosis [9, 13, 18]. However, there is an insufficiency in the literature looking at the association between arthrofibrosis and keloid formation. Therefore, it can be postulated that patients with a diagnosis of keloid formation are at an increased risk of arthrofibrosis after TKA.

With the increase in TKAs performed annually in the US, it is important to evaluate the major factors that lead to the failure of this highly utilized procedure. The purpose of this study was to identify and compare the rates of postoperative complications related to arthrofibrosis after primary TKA in patients with history of hypertrophic scar and keloid disorders versus those without, using a nationwide database to adequately power the study. Providers will be able to utilize this information to properly counsel their patients on the risks of arthrofibrotic complications.

## Materials and methods

Patient information was queried from PearlDiver (PearlDiver Inc, Fort Wayne, IN), a commercially available administrative claims database, by using the International Classification of Diseases (ICD), ninth revision and tenth revision (ICD-9/ICD-10) and current procedural technology (CPT) codes. The study made use of the Mariner dataset, containing 122 million patient records from 2007 to 2018 who were commercially insured, privately insured, or who purchased Medicare Advantage plans. This study was granted an Institutional Review Board exemption as all the data was deidentified and in compliance with the Health Insurance Portability and Accountability Act.

**Table 1:** Comparison of demographics and clinical characteristics of patients receiving primary TKA

Demographic variable	TKA w/ Hx of keloid (*n* = 11,461)	Primary TKA (*n* = 534,414)	*p*
Sex, *n* (%)			
Female	7014 (61.2)	343,482 (64.3)	0.001
Male	4447 (38.8)	199,932 (37.4)	0.042
Age, *n* (%)			
< 65	4413 (38.5)	224,759 (42.1)	< 0.001
65–79	7048 (61.5)	318,655 (59.6)	0.046
≥ 80	0 (0.0)	0 (0.0)	
BMI*, *n* (%)			
< 30	35 (0.3)	1361 (0.3)	0.333
30–40	336 (2.9)	15,307 (2.9)	0.698
≥ 40	373 (3.3)	20,905 (3.9)	< 0.001
CCI, mean ± SD	1.5 ± 2.0	1.3 ± 1.8	< 0.001
Specific comorbidities, *n* (%)			
Tobacco use	1310 (11.4)	60,994 (1.4)	0.972
Rheumatoid arthritis	562 (4.9)	23,725 (4.4)	0.024
Liver disease	737 (6.4)	28,463 (5.3)	< 0.001
Congestive heart failure	694 (6.1)	31,317 (5.9)	0.418
Cardiac disease	3003 (26.2)	126,743 (23.7)	< 0.001
COPD	2729 (23.8)	114,197 (21.4)	< 0.001
Chronic kidney disease	849 (7.4)	35,254 (6.6)	0.001
History of alcohol use	172 (1.5)	7918 (1.5)	0.900
Preoperative anemia	2250 (19.6)	90,641 (17.0)	< 0.001
Immunocompromised	319 (2.9)	13,526 (2.5)	0.104
Depression	1868 (16.3)	81,008 (15.2)	0.004
Region, *n* (%)			
South	4,944 (43.1)	210,405 (39.4)	< 0.001
Midwest	2,893 (25.2)	162,392 (30.4)	< 0.001
Northeast	2,304 (20.1)	100,760 (18.9)	0.006
West	1,311 (11.4)	69,135 (12.9)	< 0.001
90-day readmission rate, *n* (%)	978 (8.5)	39,744 (7.4)	< 0.001

*TKA* total knee arthroplasty, *BMI* body mass index, *CCI* Charlson comorbidity index, *COPD* chronic obstructive pulmonary disease

^*^BMI data were available for 9.1% of keloid TKA cases and 7.7% of primary TKA cases

**Table 2:** Comparison of postoperative complications and procedures of patients receiving primary TKA

Joint complication	TKA w/ keloid (*n* = 11,461)	Primary TKA (*n* = 534,414)	OR^a^ (95% CI)
Ankylosis, *n* (%)			
30-day	69 (0.6)	2006 (0.4)	1.65 (1.3–2.1)
90-day	347 (3.0)	14,485 (2.7)	1.17 (1.0–1.3)
6-month	475 (4.1)	19,413 (3.6)	1.19 (1.1–1.3)
1-year	593 (5.2)	22,890 (4.3)	1.26 (1.2–1.4)
Manipulation under anesthesia, *n* (%)			
30-day	23 (0.2)	1,009 (0.2)	1.1 (0.7–1.6)
90-day	393 (3.4)	1, 256 (3.2)	1.1 (1.0–1.2)
6-month	495 (4.3)	21,421 (4.0)	1.1 (1.0–1.2)
1-year	528 (4.6)	22,257 (4.2)	1.2 (1.1–1.3)
Lysis of adhesions*, *n* (%)			
90-day	13 (0.1)	299 (0.1)	2.2 (1.2–3.6)
6-month	33 (0.3)	794 (0.2)	2.0 (1.4–2.8)
1-year	54 (0.5)	1,361 (0.3)	1.9 (1.4–2.5)
Prosthetic revision, *n* (%)			
6-month	42 (0.4)	2,283 (0.4)	0.9 (0.6–1.2)
1-year	77 (0.7)	3,999 (0.8)	0.9 (0.7–1.2)
2-year	141 (1.2)	6,442 (1.2)	1.1 (0.9–1.3)

*TKA* total knee arthroplasty, *OR* odds ratio, *CI* confidence interval

^a^Adjusting for sex, age, BMI, diabetes, tobacco use, and CCI

^*^Data for 30-day lysis of adhesions not available

A retrospective cohort design was used to compare primary TKA rates in patients with a history of hypertrophic scar formation and those without. Patients receiving primary total knee arthroplasty were identified using the CPT code (27447). Patients with a diagnosis of hypertrophic scars and keloids were identified using ICD-9 diagnosis codes (7014) and ICD-10 diagnosis codes (L730, L910, L905), and were either included or excluded from the primary TKA groups to create the two patient cohorts. Patients were excluded if they received TKA due to pathologic or traumatic fracture, as well as revision TKA miscoded as primary. Additionally, patients who received a TKA on the contralateral leg were excluded to ensure outcomes were related to the primary TKA under investigation. Only patients who underwent primary TKA between 2010 and the second quarter of 2016 (up to and including June 30th) were included to ensure a minimum 2-year follow up in the database for all included patients. The CPT and ICD codes defining the patient groups are located in Appendix Table 3.

The two cohorts were then queried for common diagnoses and procedures following primary TKA including ankylosis, MUA, LOA, and revision TKA. Patients developing ankylosis, undergoing MUA, or having LOA were identified using ICD diagnosis codes and queried if diagnosis occurred within 30 days, 90 days, 6 months, and 1 year following TKA. Patients who underwent revision TKA were included if revision occurred within 6 months, 1 and 2 years postoperatively of primary TKA. The CPT and ICD codes defining the patient groups are located in Appendix Table 4.

Both patient cohorts were queried for demographic information, hospital region, clinical characteristics, and hospital course data including age, sex, body mass index (BMI), Charlson comorbidity index (CCI), cost, and incidences of several specific comorbidities. Regional data were categorized using the United States Census Bureau classification of Northeast, South, West, and Midwest. Specific comorbidities queried from the database included a history of diabetes, hypertension, chronic kidney disease, congestive heart failure, coronary artery disease, rheumatoid arthritis, liver disease, immunocompromised status, history of tobacco or alcohol use, obesity, depression, other cardiac disease, preoperative anemia, and chronic obstructive pulmonary disease (COPD). An immunocompromised status was defined as receiving an immunologic agent or antineoplastic drug within one year prior to the index procedure. “Other cardiac disease” was delineated by a prior diagnosis of coronary or ischemic heart disease.

Data analysis was performed using R statistical software (R Project for Statistical Computing, Vienna, Austria) that is integrated within the PearlDiver software. An *α* level below 0.05 was considered statistically significant. Categorical variables, including demographic and clinical characteristics, were compared using chi square analysis, while a Welch’s *t*-test was used to compare continuous variables such as CCI. Multivariate logistic regression was performed to identify the association between the two patient groups after adjusting for patient age, sex, CCI, BMI, diabetes status, and alcohol or tobacco use. This regression was used to calculate odds ratios (ORs) and corresponding 95% confidence intervals (CIs) for the rates of local and systemic complications between patients with and without a history of hypertrophic scars.

## Results

**Fig. 1:**
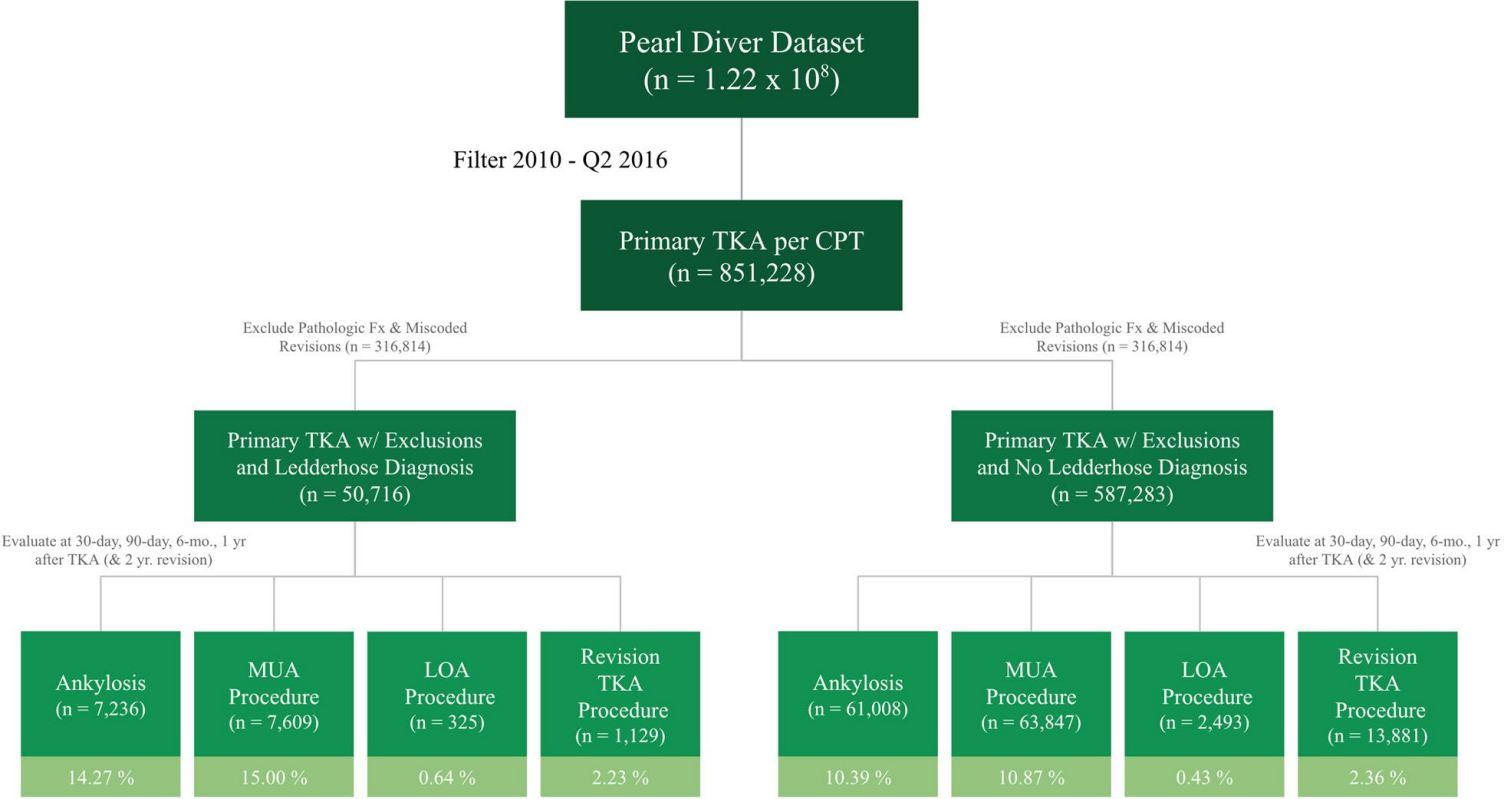
Creation of primary patient cohorts with ankylosis and procedures following primary TKA

A total of 851,228 patients undergoing primary TKA between 2010 and the second quarter of 2016 were queried from the PearlDiver database using CPT codes. After adjusting for exclusion criteria and dates for appropriate follow-up procedure, this number decreased to 545,875. Of these patients, 11,461 (2.1%) had a keloid diagnosis at any time point in their record, and 534,414 (97.9%) did not (Fig. 1). Table 1 shows a greater proportion of patients undergoing primary TKA with keloid diagnosis were male (male 38.8 versus 37.4%, *p* 0.042), between the ages of 65–79 (61.5 versus 59.6%, *p* 0.046), were less likely to have a BMI classification greater than 40 (3.3 versus 3.9%, *p* < 0.001) and had a higher average burden of comorbidities (CCI: 1.5 versus 1.3, *p* < 0.001). TKA patients in the keloid diagnosis cohort had higher rates of these specific comorbidities: rheumatoid arthritis, 4.9 versus 4.4%, *p* = 0.024; liver disease, 6.4 versus 5.3%, *p* < 0.001; cardiac disease, 26.2 versus 23.7%, *p* < 0.001; COPD, 23.8 versus 21.4%, *p* < 0.001; CKD, 7.4 versus 6.6%, *p* 0.001; preoperative anemia, 19.6 versus 17.0%, *p* < 0.001; and depression, 16.3 versus 15.2%, *p* 0.004. With regards to US regions, the percentage of TKAs performed on patients with keloid diagnosis in the South and Northeast were larger compared with the primary TKA cohort with no keloid diagnosis (South 43.1 versus 39.4%, *p* < 0.001; Northeast 20.1 versus 18.9%, *p* 0.006), and lower in the West and Midwest (West 11.4 versus 12.9%, *p* < 0.001; Midwest 25.2 versus 30.4%, *p* < 0.001). The 90-day readmission rate between the two was also greater in the keloid diagnosis group (8.5 versus 7.4%, *p* < 0.001).

Patients undergoing TKA with keloid diagnosis had a significantly higher association with Ankylosis within 30 days (OR, 1.7; 95% CI, 1.3–2.1), 90 days (OR, 1.2; 95% CI, 1.0–1.3), 6 months (OR, 1.2; 95% CI, 1.1–1.3), and 1 year (OR, 1.3; 95% CI, 1.2–1.4) following primary TKA (Table 2). Primary TKA for patients with keloids also had a significantly greater association with MUA **(**90-day OR, 1.1; 95% CI, 1.0–1.2; 6-month OR, 1.1; 95% CI, 1.0–1.2; 1-year OR, 1.2; 95% CI, 1.1–1.3) and LOA (90-day OR, 2.2; 95% CI, 1.2–3.6; 6-month OR, 2.0; 95% CI, 1.4–2.8; 1-year OR, 1.9; 95% CI, 1.4–2.5). Finally, with regards to revision TKA, there was no significant difference found between the two patient cohorts seen at 6 months and 1 and 2 years.

## Discussion

The present study indicates patients who have a diagnosis of keloids are at an increased odds risk of arthrofibrosis and the subsequent procedures necessary to manage this complication. Patients with keloids had a significantly higher rate of being diagnosed with ankylosis at all observed time periods post TKA. The literature is lacking on whether there exists an increased risk of developing arthrofibrosis following TKA and keloid formation; however, the current findings support that a relationship exists between the development of arthrofibrosis and a diagnosis of keloid. Additionally, this study demonstrated patients with a diagnosis of keloids underwent MUA and LOA at a significantly higher rate. Although the keloid cohort had a higher rate of arthrofibrosis diagnosed compared with the no-keloid cohort, there was no significant difference in the rate of revision TKA among these cohorts at any observed time period. This observation is likely multifactorial, but could be in part due to the short 2-year follow up utilized in this study, as well as other common causes of TKA failure such as infection, instability, and aseptic loosening [19, 20].

The etiologies of keloids and arthrofibrosis are not fully understood, however both pathologies are hypothesized to be associated with an error within the fibrotic cascade resulting from over-expression of transforming growth factor β [9, 21]. With regards to keloids, the incidence has been significantly higher in ethnicities of darker complexion, with individuals of African and Asian decent being most at risk [22].

Previous studies have reported arthrofibrosis occurring at a rate of approximately 5% of patients undergoing TKA [9, 20]. Those findings align with the results of this study where the keloid cohort had a significantly higher rate of arthrofibrosis (5.2%) than the primary TKA cohort (4.3%). It is important to note that arthrofibrosis is documented as one of the major causes of TKA failure, with multiple studies reporting it as the causative agent of roughly 2 → 14% of TKA failures [19, 20, 24]. With an increased risk of arthrofibrosis after TKA in patients having a diagnosis of keloid and hypertrophic scars, the potential for future procedures and reoperations to treat the arthrofibrosis also increases. While timing of MUA is controversial, the general consensus agrees that MUA within the first 12-weeks of operation is the treatment of choice if the patient fails to achieve greater than 90° of flexion with physical therapy [25, 26]. Issa et al. also demonstrated early MUA within 12 weeks postoperatively if flexion is < 90° to gain the greatest amount of knee range of motion and higher clinical outcomes [26]. Enad et al. proposed an algorithm recommending patients receive a MUA within 3 months postoperatively, after which they recommend including lysis of adhesions [27]. LOA needs to be considered in patients who failed to achieve acceptable range of motion after therapy and MUA [28, 29]. Furthermore, if MUA and LOA are unsuccessful in treating the stiffness associated with arthrofibrosis, then the option of revision TKA should be considered. In a retrospective review, Rutherford et al. found revision TKAs for arthrofibrosis had significant improvement in range of motion in both flexion and extension [30].

Surgeons should consider increased posterior slope in cruciate retaining knees and pay close attention not to over tighten in flexion for the potential contractures in patients with keloids and hypertrophic scars [31–33]. Ouellet et al. demonstrated the most intensive rehabilitation program should occur in the first months following TKA for optimal results [34]. Furthermore, the utilization of dynamic bracing has been shown to significantly increase patients range of motion when used early in the postoperative period following TKA and could also be used to reduce incidence and severity of stiffness if patients are lagging in flexion/extension gains in their postoperative course [35]. While current guidelines and practices for follow up after TKA vary widely, it has been demonstrated that early knee range of motion predicts longer term range and suggests a benefit of increasing frequency of follow-up visits for at-risk patients to monitor for potential contracture [36, 37]. In addition, Liveberg et al. documented patients who received a preoperative informational meeting on the procedure and expectations following TKA had a decreased risk of arthrofibrosis [38]. Increased awareness in both the patient and physician should prompt discussion of expected range of motion in the postoperative period along with early and aggressive physical therapy.

An inherent limitation of this administrative claims database study is that the accuracy of the findings depends on the correct selection of codes in the database, which is subject to human error. This is mitigated by the large number of patients included in the study and limits the potential for entry error that may cause significant fluctuations in results. Additionally, because this study included patient data prior to and after 2015, the diagnosis/procedural codes were not exact matches across ICD-9 and ICD-10. To address this lack of continuity between ICD-9 and ICD-10 codes, a code translator was used to identify corresponding codes. Clinical data such as race, prior surgery to the knee undergoing TKA, blood loss, implant type, patient outcome scores, and radiographic images could not be queried from the database; however, they likely would have influenced the outcomes demonstrated in this study. While confounders were reduced with the use of multivariate logistic regression, it is possible that other confounders influenced the data. Revisions were only accounted for up to 2 years postoperatively in order to maximize the total amount of patients included in the study.

## Conclusion

Patients with keloids have an increased odds risk of arthrofibrosis following primary TKA. These patients are subsequently at a higher risk of undergoing the procedures necessary to treat arthrofibrosis such as MUA and LOA. Early and aggressive physical therapy, dynamic bracing, and both physician and patient awareness should be considered in this cohort to improve outcomes. Surgeon awareness could also help guide decisions on intraoperative gap balancing to achieve optimal results. Future studies investigating confounding factors such as race, prior surgery, range of motion, and postoperative recovery are needed to determine the association of keloid formation and subsequent arthrofibrosis following primary TKA.

## Data Availability

All data generated or analyzed during this study are included in this published article and its supplementary information files.

## Appendix Codes Used to Identify Patients and Procedures

See Tables 3 and 4.

**Table 3:** Codes used to evaluate for TKA and hypertrophic scarring

TKA		
CPT-27447		
Hypertrophic scar formation		
ICD-9-D-7014	ICD-10-D-L730	ICD-10-D-L910
ICD-10-D-L905		
Miscoded revision TKA		
ICD-10-P-0SPC08Z	ICD-10-P-0SPD08Z	ICD-10-P-0SPT0JZ
ICD-10-P-0SPC09Z	ICD-10-P-0SPD09Z	ICD-10-P-0SPT4JZ
ICD-10-P-0SPC0JC	ICD-10-P-0SPD0JC	ICD-10-P-0SPU0JZ
ICD-10-P-0SPC0JZ	ICD-10-P-0SPD0JZ	ICD-10-P-0SPU4JZ
ICD-10-P-0SPC38Z	ICD-10-P-0SPD38Z	ICD-10-P-0SPV0JZ
ICD-10-P-0SPC3JZ	ICD-10-P-0SPD3JZ	ICD-10-P-0SPV4JZ
ICD-10-P-0SPC48Z	ICD-10-P-0SPD48Z	ICD-10-P-0SPW0JZ
ICD-10-P-0SPC4JZ	ICD-10-P-0SPD4JZ	ICD-10-P-0SPW4JZ
Exclusion codes		
ICD-9-D-73315 ICD-9-D-73397 ICD-9-D-82100 ICD-9-D-82110 ICD-9-D-82120 ICD-9-D-82123 ICD-9-D-82129 ICD-9-D-82130 ICD-9-D-82132 ICD-9-D-82133 ICD-9-D-82139 ICD-9-D-73316 ICD-9-D-73393 ICD-9-D-82300 ICD-9-D-82302 ICD-9-D-82310 ICD-9-D-82312 ICD-9-D-82380 ICD-9-D-82382 ICD-9-D-82390 ICD-9-D-82392 ICD-9-P-0080	ICD-9-P-0081 ICD-9-P-0082 ICD-9-P-0083 ICD-9-P-0084 ICD-9-P-8155 ICD-9-P-8006 ICD-10-D-M84453A ICD-10-D-M84750A ICD-10-D-M84353A ICD-10-D-S7290XA ICD-10-D-S7290XB ICD-10-D-S7290XC ICD-10-D-S72409A ICD-10-D-S72453A ICD-10-D-S72456A ICD-10-D-S72499A ICD-10-D-S72409B ICD-10-D-S72453B ICD-10-D-M84469A ICD-10-D-M84369A ICD-10-D-S82109A	ICD-10-D-S82101A ICD-10-D-S82831A ICD-10-D-S82102A ICD-10-D-S82832A ICD-10-D-S82109B ICD-10-D-S82109C ICD-10-D-S82101B ICD-10-D-S82831B ICD-10-D-S82102B ICD-10-D-S82832B ICD-10-D-S82201A ICD-10-D-S82401A ICD-10-D-S82202A ICD-10-D-S82402A ICD-10-D-S82201B ICD-10-D-S82201C ICD-10-D-S82401B ICD-10-D-S82202B ICD-10-D-S82402B ICD-10-P-0SPC0JZ ICD-10-P-0SPD0JZ

Excluded patients with TKA due to traumatic or pathologic fracture and revision TKA

*TKA* total knee arthroplasty, *CPT* current procedural terminology, *ICD-9/ICD-10* International Classification of Diseases, ninth and tenth revision, *CMS* Centers for Medicare and Medicaid Services

^a^ICD-10 codes were retrieved via combination mapping from related ICD-9 codes according to CMS guidelines

**Table 4:** Codes used to evaluate for ankylosis, manipulation under anesthesia, lysis of adhesions, and revision TKA

Ankylosis		
ICD-9-D-71859	ICD-9-D-71858	ICD-9-D-71856
ICD-9-D-71850	ICD-10-D-M2460	ICD-10-D-M24661
ICD-10-D-M24662	ICD-10-D-M24669	
MUA		
CPT-27570		
LOA		
CPT-29884		
Revision TKA		
CPT-27486	CPT-27487	

*ICD-9/ICD-10* International Classification of Diseases, ninth and tenth revision, *MUA* manipulation under anesthesia, *CPT* current procedural terminology, *LOA* lysis of adhesions; *TKA* total knee arthroplasty

^a^ICD-10 codes were retrieved via combination mapping from related ICD-9 codes according to CMS guidelines

## Abbreviations

TKA: Total knee arthroplasty
MUA: Manipulation under anesthesia
LOA: Lysis of adhesions
ICD: International Classification of Diseases
CPT: Current procedural technology
OR: Odds ratio
CI: Confidence interval
BMI: Body mass index
CCI: Charlson comorbidity index
COPD: Chronic obstructive pulmonary disease
